# Preserved periprosthetic bone stock at 5 years post-operatively with uncemented short hip stem in both collared and collarless version

**DOI:** 10.1007/s00402-021-04225-z

**Published:** 2021-11-29

**Authors:** Ola Belfrage, Erik Weber, Martin Sundberg, Gunnar Flivik

**Affiliations:** Department of Orthopedics, Skåne University Hospital and Clinical Sciences, Lund University, Lund, Sweden

**Keywords:** THA, DXA, Short stem THA, Bone density

## Abstract

**Introduction:**

Previous bone density studies have generally shown bone resorption around both cemented and uncemented total hip arthroplasty (THA) stems. This is presumed to be due to stress shielding. Short stems have been introduced partly to preserve bone in the proximal femur by a more physiological loading of the bone. The purpose of this study was to evaluate bone remodeling around a short, fully hydroxyapatite-coated titanium stem that comes in a collared and collarless version.

**Patients and methods:**

A prospective cohort of 50 patients included in a study evaluating the Furlong Evolution stem has been followed for 5 years. Examination was done with dual energy X-ray absorptiometry (DXA) postoperatively, at 1, 2 and 5 years. Clinical outcome was followed with radiography and both general and hip specific outcome measures.

**Results:**

The two versions of the stem behaved similarly regarding bone remodeling. After an initial decrease up to 1 year, bone mineral density (BMD) increased in all Gruen zones up to 2 years and at 5 years bone stock was still preserved compared with postoperatively (net BMD + 1.2% (95% CI − 0.4 to 2.8)). Increase in BMD occurred mainly in the greater trochanter and distally around the stem with a decrease in the calcar area. Both versions showed excellent clinical outcome up to 5 years.

**Conclusion:**

This short stem seems to preserve proximal bone stock up to 5 years, exhibiting similar behaviour both with and without a collar.

**Trial registration number and date of registration:**

ClinicalTrials.gov, (identifier: NCT01894854). July 10, 2013.

## Introduction

Implantation of a femoral stem gives rise to an altered mechanical distribution of the forces on the proximal femur. After surgery, according to Wolff’s law, the bone in the proximal femur will tend to grow thicker where the bone is loaded, and osteopenic where the bone is less loaded compared with before surgery [1]. With a long and inflexible uncemented femoral stem with primarily diaphyseal anchoring, the forces bypass the metaphyseal bone leaving it less loaded, resulting in resorption in the proximal femur due to stress shielding [2]. Therefore, there has been a growing interest in the orthopaedic community regarding short femoral stems, which could anchor the stem in the metaphysis instead of the diaphysis. A shorter stem will involve a lesser part of the femoral bone where stress shielding can occur. Studies of proximally loading stems have shown a smaller decrease in bone mineral density (BMD) in the proximal femur compared with conventional uncemented stems [3–5]. Typical dual energy X-ray absorptiometry (DXA) findings display a reduction in bone density around the prosthesis. This is most noticeable in the immediate post-operative period, after which a rebound can occur with a recovery in bone density [6, 7]. In the calcar area, Gruen zones 6 and 7, there will usually be a residual reduction in bone density [2].

**Fig. 1:**
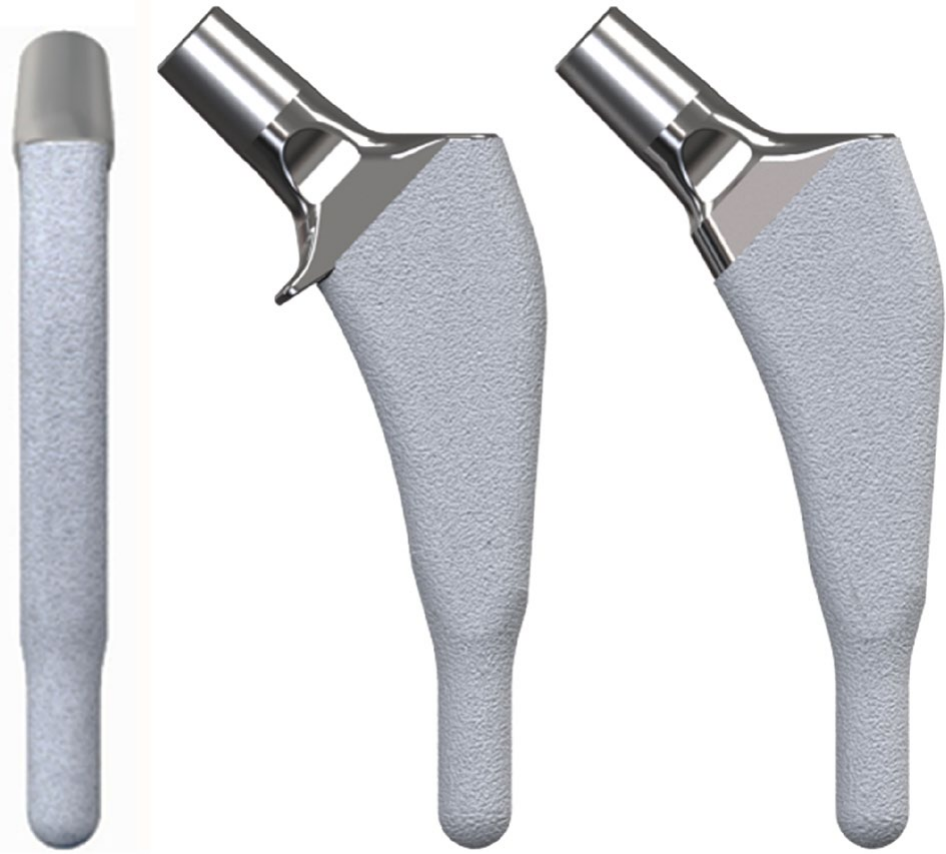
Stem design in lateral view, the collared and the collarless version with 133-degree neck shaft angle

The Furlong Evolution (JRI Orthopaedics Ltd., Sheffield, UK), introduced in 2011, is a short, fully hydroxyapatite-coated (HA-coated), uncemented stem in titanium alloy (Ti-6Al-4 V). It comes in a collared and collarless version (Fig. 1). The stem design is based on its precursors: the Furlong HAC [8], and the later introduced Furlong Active [9], but with some new design features intended to promote metaphyseal anchoring. The diaphyseal component of the stem is shortened; all sizes are 100 mm long. The shoulder is less prominent than its precursors and the roughness of the coating is more prominent proximal to the distal cylindrical part of the stem. The stem is available in two different offset versions and in two different neck shaft angles (126° and 133°).

The optional collar is not a classic type of collar; it is, rather, a small “lip” resting on or close to the resected neck. It is designed to prevent excess post-operative subsidence. The collar can be visualized only in the AP view, and not in the medio-lateral view (Fig. 1).

In this study, designed to comprise both DXA and radiostereometry (RSA), we wanted to examine the BMD over time around this new short stem hip arthroplasty and to compare the two different versions of the stem: the collared versus the collarless. Our hypothesis was that the collar would increase the loading of the bone in the calcar region and reduce the bone loss in this area. Here we present the results from the DXA examinations up to 5 years.

## Patients and methods

### Study group

Fifty patients (50 hip joints, 34 men) with a mean age of 60 years (range 36–75) with primary osteoarthritis scheduled for total hip arthroplasty (THA) at Skåne University Hospital were recruited and enrolled for surgery between September 2012 and June 2013 (Table 1). The exclusion criteria were Charnley category C patients, patients with a femur anatomy which on plain radiographs was considered to be unsuitable for an uncemented stem, previous fracture or operation to the hip, rheumatoid arthritis, malignant disease, ongoing corticosteroid or immunosuppressive medication, dementia, drug or alcohol abuse.

**Table 1:** Patient baseline characteristics

Variables	Collar	Collarless	Total
No. of patients	25	25	50
Age (range)	58.5 (36–75)	60.2 (43–75)	59.8 (36–75)
Gender (M = male; F = female)	16 M; 9F	18 M; 7F	34 M; 16F
BMI (SD)	26.7 (3.2)	26.6 (3.3)	26.6 (3.2)

**Fig. 2:**
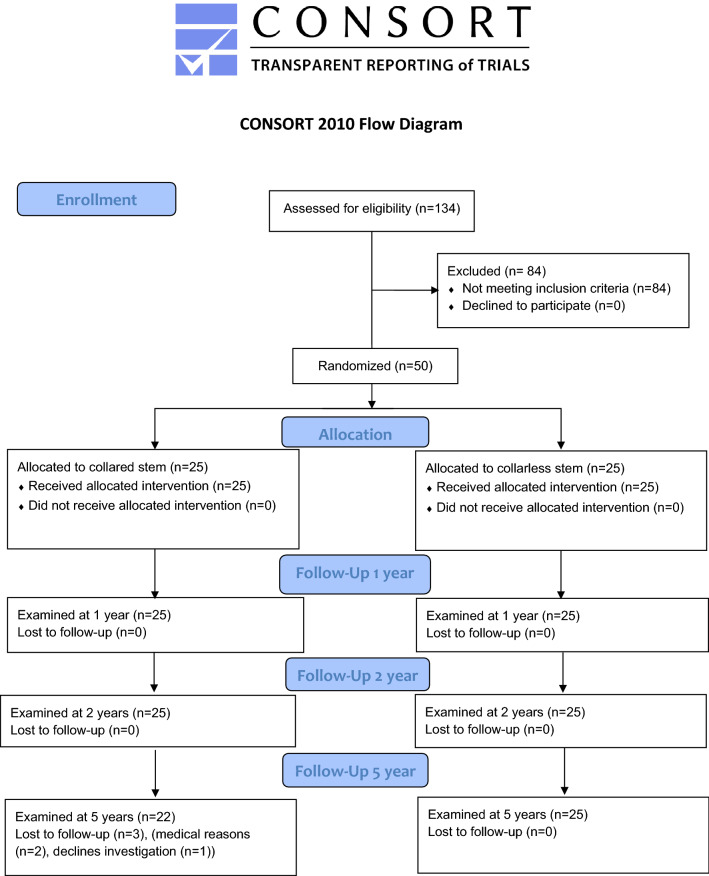
CONSORT flow diagram

A blocked randomization order, created by statistics software, was used and put into closed envelopes that were opened intra-operatively, one at each operative procedure. 25 patients were thus randomized to receive a collared stem, and 25 patients received a collarless stem. No patients were excluded from the study. All 50 patients initially included were examined at 1 and 2 years, and 47 at 5 years (Fig. 2).

### Surgery

The surgical procedures were performed through a postero-lateral approach and were carried out by two experienced hip surgeons (GF, MS). All patients received a CSF *Plus* uncemented acetabular cup and a 32 mm femoral head (JRI Orthopaedics Ltd., Sheffield, UK). All patients received standard peri-operative antibiotics (cloxacillin) and post-operative prophylaxis against venous thrombosis (enoxaparin). Post-operatively the patients were fully mobilized with the aid of two crutches and were allowed full weight bearing.

### DXA examinations

To evaluate post-operative BMD, all patients underwent examinations using a GE Lunar Prodigy DXA scanner (GE Healthcare, Chicago, IL, USA) within 2 weeks of surgery as a baseline examination. The patient was placed in a supine position, with the legs extended and the foot of the operated side held in a neutral position by a positioning device, for a standardized neutral position of the hip joint. The follow-up DXA examinations were conducted at 1 year, 2 years and 5 years with a time tolerance of ± 5% at each examination. BMD was computed for all seven Gruen zones. Net BMD, a pooled value for all seven Gruen zones, was calculated as total bone mineral content divided by the total area. Double examinations were performed on all patients except one during the follow-up period and the precision error of the DXA examinations was calculated [10] (Table 2).

**Table 2:** Precision error of DXA measurement analyses in the seven Gruen zones expressed as the coefficient of variation in percentage, obtained from 49 double examinations [10]

Gruen zones	Precision error (% CV)
1	0.34
2	0.66
3	0.79
4	0.32
5	0.78
6	0.79
7	0.99

### Clinical and radiographic follow-up

Routine clinical follow-ups were performed at 3, 12 and 24 months. Standard radiographic examinations were performed pre-operatively, on the first post-operative day, then at 12 and 24 months. RSA examinations were performed according to protocol. The patients completed the self-administered Hip disability and Osteoarthritis Outcome Score (HOOS) [11] and the general health questionnaire EQ-5D prior to surgery, at one, two and five years’ follow-up.

### Statistics

The primary effect variable was RSA migration and 50 patients; 25 patients in each group were included in the study based on a power calculation derived from expected RSA data. These results have been published previously [12]. The secondary DXA outcome measure was difference in bone density changes as measured in the seven Gruen zones at one year. The patients have now been followed up for up to 5 years with DXA and analysis of difference in net BMD up to 5 years has been added.

According to Shapiro–Wilks and Q–Q plots, the BMD data were found to be sufficiently normally distributed; thus the 95% confidence intervals (CI) of the examinations are presented symmetrically around the mean. To compare differences between groups at a given time, Student’s t-test was used. A general linear mixed-model analysis was used to determine the change in bone density over time. For analysis of the outcome questionnaires HOOS and EQ-5D, we used the Mann–Whitney U-test to compare differences between the groups at a given time.

We used the IBM SPSS statistics software version 25.0 (IBM, Armonk, NY, USA) and SAS Enterprise Guide Version 6.100.0.4025 (SAS Institute, Cary, IN, USA). A *p* value < 0.05 was considered significant.

### Ethics and registration

The study was approved by the Ethical Committee of Lund University (Dnr: 2012/53). All patients gave their informed written consent. The study was carried out in compliance with the Helsinki Declaration of 1975, as revised in 2000, and was registered at ClinicalTrials.gov (identifier: NCT01894854).

## Results

In the collarless group, all patients were examined up to 5 years post-operatively. In the collared group three patients declined examination at 5 years, all three as a result of conditions not related to the hip. Their data have been analysed for 2 years.

There were no statistically significant differences between the groups regarding baseline BMD or net BMD at 1, 2 or 5 years. The collar seemed to have minimal influence on bone remodelling around the stem, even in the calcar area (Table 3).

**Table 3:** Change in bone mineral density (BMD) in the seven Gruen zones and combined net value. Numbers are % difference in mean versus post-operative examination with 95% confidence interval

Gruen zone	Comparison vs. baseline	Change in BMD	95% CI
Zone 1	1 year	− 1.5	− 6.4 to 3.4
	2 year	5.4	0.8 to 10.0
	5 year	3.9	0.1 to 7.7
Zone 2	1 year	− 10.1	− 14.0 to − 6.2
	2 year	− 1.8	− 5.5 to 1.8
	5 year	− 5.3	− 8.4 to − 2.2
Zone 3	1 year	1.2	− 1.3 to 3.7
	2 year	7.3	5.0 to 9.5
	5 year	7.8	6.0 to 9.7
Zone 4	1 year	− 2.9	− 4.4 to − 1.5
	2 year	0.8	− 0.6 to 2.2
	5 year	1.2	0.1 to 2.3
Zone 5	1 year	2.0	0.0 to 4.0
	2 year	8.7	6.9 to 10.6
	5 year	9.1	7.6 to 10.6
Zone 6	1 year	− 9.2	− 11.6 to − 6.7
	2 year	− 6.0	− 8.4 to − 3.5
	5 year	− 8.7	− 11.3 to − 6.1
Zone 7	1 year	− 15.2	− 19.2 to − 11.2
	2 year	− 10.4	− 14.4 to − 6.4
	5 year	− 15.9	− 20.3 to − 11.6
Net BMD	1 year	− 2.9	− 4.4 to − 1.5
	2 year	2.3	0.8 to 3.7
	5 year	1.2	− 0.4 to 2.8

**Fig. 3:**
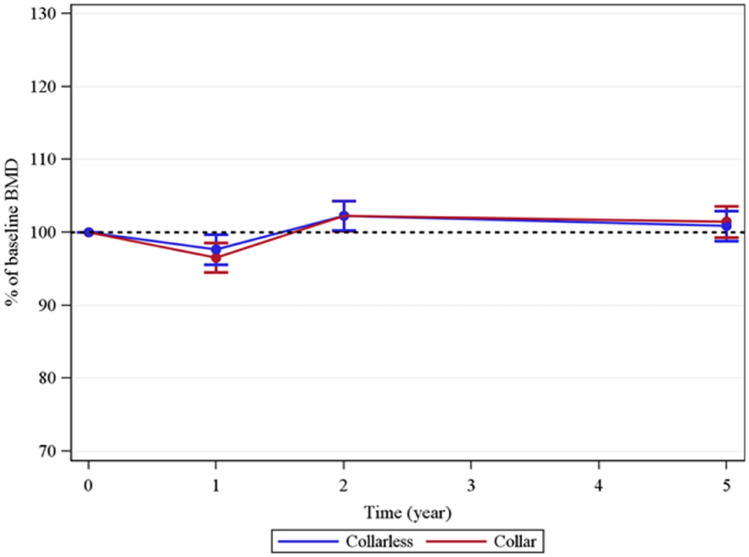
Net BMD vs. time

At one year there was a decrease in net BMD, − 2.9% (95% CI − 4.4 to − 1.5). Between 1 and 2 years, an increase occurred in all seven Gruen zones. At 2 years, the net BMD had increased compared with baseline BMD with a mean of 2.3% (95% CI 0.8–3.7). Between 2 and 5 years, differences were small with a slight, statistically insignificant, decrease in net BMD mainly due to bone resorption in the calcar region (Fig. 3).

**Fig. 4:**
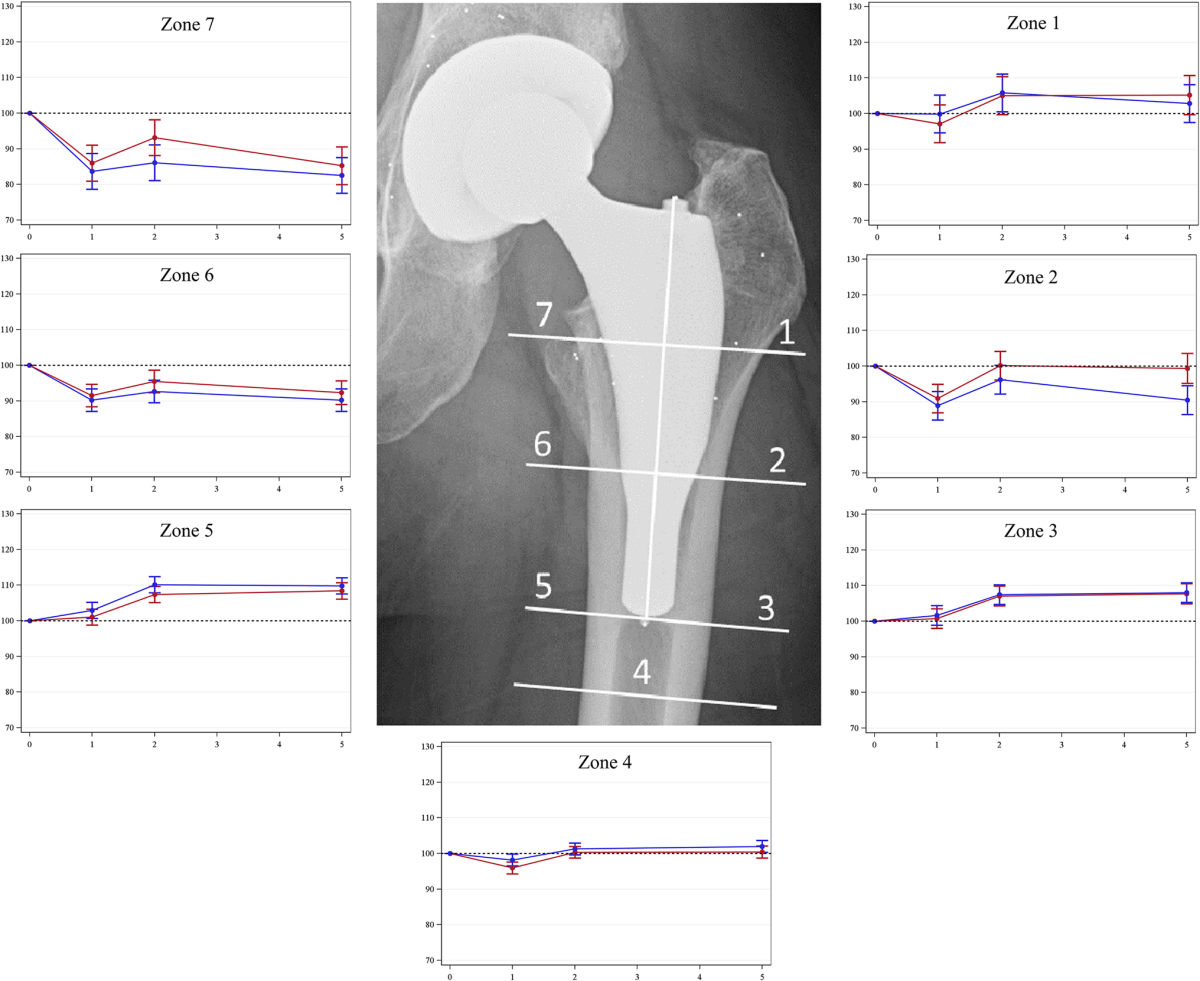
Radiograph demonstrating the seven Gruen zones. Graphs represent the change in BMD in percentage (y-axis) over time in years (x-axis). Blue = collarless; red = collared

Medially, in the calcar area, BMD decreased slightly for both stem types over the study period. More distally around the stem, BMD had increased significantly at 2 years and this difference remained at 5 years. In the greater trochanteric region, there were minor changes in BMD and the same applies to the area distal to the stem (Fig. 4).

There were no serious adverse events during the 5-year follow-up period. No stems have been revised or considered to be loose based on RSA or standard radiological examinations.

HOOS and EQ-5D displayed similar results in both groups pre-operatively and throughout follow-up, with no statistical difference between the groups. There was an anticipated marked improvement in both groups when comparing pre-operative results of both EQ-5D and HOOS scores with the post-operative results at 1, 2 and 5 years.

## Discussion

In this cohort of 50 patients we found similar bone remodelling around the Furlong Evolution short-stem prosthesis with or without a collar. For up to 5 years, the periprosthetic bone was well preserved compared with baseline values. No stems were revised.

Bone resorption is common around both cemented and uncemented femoral stems. Stress shielding, or disuse atrophy, results partly from the difference in elasticity between the implant and the periprosthetic bone. This is especially evident in longer stems with a more distal femoral anchorage [2, 13].

In our study there was a decrease in bone density during the first year. Other studies have shown that most of this resorption occurs during the first months after surgery [7, 13]. The calcar region is especially exposed to stress shielding with a decrease in bone density in most studies [3, 7, 15–17]. This also applies to short-stemmed prostheses developed to be bone sparing where an increase in BMD takes place more distally around the stem [18]. In a review article by Yan comparing four different short-stem prostheses, BMD decreased between 4.5% and 30% in Gruen zone 7 at 1 year [19]. One exception is a study on the Accolade II stem where BMD in the calcar area was unchanged at the 1-year follow-up. However, the first DXA examination (baseline) in that study was at 6 weeks post-operatively, when a large part of the resorption can already be assumed to have taken place [20].

Ageing is associated with substantial bone loss in both women and men. The menopause leads to more dramatic bone loss and after that slower age-related bone loss continues throughout life, with a reduction in BMD of almost 1% per year [21]. This natural decrease in bone density could potentially explain the results of our study with a decrease in BMD between two and five years post-operatively.

A recent registry study from the United Kingdom National Joint Registry (NJR) found a nearly five-fold increase in relative risk for early periprosthetic fractures for collarless uncemented stems compared with collared stems. This is explained by the collar increasing compressive load on the cortical bone during rotational injury [22]. The collar on this prosthesis is a small lip that is visible only in one plane. The advantage of a small collar as compared with a larger one is to minimize the risk of iliopsoas impingement, which has been described [23]. Its main purpose is to reduce excessive post-operative subsidence. We hypothesised that it would also decrease bone resorption in the calcar area due to increased cortical load. To achieve this, contact between the collar and calcar needs to be obtained during surgery or in the initial settling-in phase. In our study, we found collar–calcar contact on post-operative X-rays in 16 of 25 cases (64%), which is in line with other published studies [24]. The stems are expected to subside in the initial post-operative period so some lips may have established contact after a couple of weeks while other lips remained inactive. The difference between groups in bone density was very small and the fact that not all collars were active immediately post-operatively may have contributed to the study being underpowered to detect a possible real difference in bone density. There were no periprosthetic fractures in our study, which we had not expected anyway given that the incidence of this complication is very low [22]. The theoretical increase in risk with a collarless stem can speak to the advantage of a collared stem. According to earlier published RSA data there were no significant difference between the two stem types regarding migration. Both had stabilised at three months and thereafter showed consistent almost steady state position, interpreted as good osseointegration [12]. This indicates that the lip does not have a major impact on either stem migration or bone density. However, none of our results contradict the use of the collared stem and in individual patients it may make a difference.

DXA is considered the most reliable method of measuring BMD after total hip arthroplasty [3, 15, 25]. In this study, on a short-stem prosthesis Gruen zone 7 becomes very small, which is reflected in the largest precision error of the measurements. We believe that this study contains a sufficient number of patients to evaluate the behaviour of this stem concept regarding periprosthetic bone adaptation. A strength of the study is the 5 years’ follow-up with only three patients lost to follow-up, and no revisions or major complications.

## Conclusion

In conclusion, this shorter and modified prosthesis based on a previously well-proven concept seems to preserve proximal bone stock up to 5 years post-operatively with no revisions or major complications in this series of 50 patients. The collared and the collarless versions behaved similarly. Further assessment and close monitoring is advocated in the introduction of a novel implant. Long-term survivorship could be the scope of further investigations.

## Data Availability

Researchers from the scientific community may request access to the anonymised patient-level data from our study by providing a scientific research proposal.
